# Multi-Criteria Optimisation of Friction Stir Welding Parameters for EN AW-2024-T3 Aluminium Alloy Joints

**DOI:** 10.3390/ma15155428

**Published:** 2022-08-07

**Authors:** Andrzej Kubit, Tomasz Trzepieciński, Rafał Kluz, Krzysztof Ochałek, Ján Slota

**Affiliations:** 1 Department of Manufacturing and Production Engineering, Rzeszow University of Technology, al. Powst. Warszawy 8, 35-959 Rzeszów, Poland; 2 Department of Mechanical Engineering, State School of Higher Vocational Education, Rynek 1, 38-400 Krosno, Poland; 3 Institute of Technology and Materials Engineering, Technical University of Košice, Mäsiarska 74, 040 01 Košice, Slovakia

**Keywords:** EN AW-2024-T3, aluminium alloy, friction stir welding, optimisation, welding parameters

## Abstract

The aim of this research was the selection of friction stir welding (FSW) parameters for joining stiffening elements (Z-stringers) to a thin-walled structure (skin) made of 1 mm-thick EN AW-2024 T3 aluminium alloy sheets. Overlapping sheets were friction stir welded with variable values of welding speed, pin length (plunge depth), and tool rotational speed. The experimental research was carried out based on a three-factor three-level full factorial Design of Experiments plan (DoE). The load capacity of the welded joints was determined in uniaxial tensile/pure shear tests. Based on the results of the load capacity of the joint and the dispersion of this parameter, multi-criteria optimisation was carried out to indicate the appropriate parameters of the linear FSW process. The optimal parameters of the FSW process were determined based on a regression equation assessed by the Fisher–Senecor test. The vast majority of articles reviewed concern the optimisation of welding parameters for only one selected output parameter (most often joint strength). The aim of multi-criteria optimisation was to determine the most favourable combination of parameters in terms of both the smallest dispersion and highest load capacity of the joints. It was found that an increase in welding speed at a given value of pin length caused a decrease in the load capacity of the joint, as well as a significant increase in the dispersion of the results. The use of the parameters obtained as a result of multi-criteria optimisation will allow a minimum load capacity of the joints of 5.38 kN to be obtained with much greater stability of the results.

## 1. Introduction

Friction Stir Welding (FSW) is a solid-state joining technique that uses frictional heat generated by a rotating tool to join similar or dissimilar materials. FSW was invented and experimentally proven at The Welding Institute (TWI) in 1991. Currently, many variations of the FSW process have been developed, i.e., Friction Stir Spot Welding [1], Pinless Friction Stir Spot Welding [2,3], Swing Pinless Friction Stir Spot Welding [4], Ultrasonic-assisted Friction Stir Spot Welding [5], Refill Friction Stir Spot Welding [6].

Various metals which are considered non-weldable in other welding processes, for example, resistance welding, can be joined using FSW. Using this method, parts can be constructed from two different materials and thus the production costs can be significantly reduced. The heat-affected zone (HAZ) within the joint is very narrow and fine-grained and has a smooth transition zone to the base material (BM). FSW is an environmentally friendly process where there is no need for fillers, fluids, or shielding gases compared to conventional electrical, gas, and resistance welding processes [7,8].

Various theories have been described in the literature [9] explaining the formation of joints during solid-state joining. The most important of these are the adhesive, recrystallisation, and energy hypotheses [9,10]. According to the adhesive theory, pressure is applied to the welded elements in order to increase the contact area and this causes plastic deformation of the metal. Plastic deformation is accompanied by the deterioration of the surface layer and thus an increase in its free energy. According to the recrystallisation hypothesis, metals are joined thanks to the growth of new grains at the expense of the grains of both the materials that are joined. According to the energy hypothesis, if the grains of the bonded elements touch in such a way that their micro contacts have a consistent crystallographic orientation, then less energy should be used to join them [10]. In the activated areas that occur in the joining zone, larger and larger areas of mutual interaction are formed in which metallic bonds are formed [9]. It should be noted here that the FSW process is treated as a non-diffusion process.

Due to the lack of a need to use an additional material, which increases the weight of the structure and the possibility of welding aluminium alloys of the 2xxx, 6xxx, and 7xxx series [11,12], FSW is increasingly used in the aircraft industry. The FSW method was used for the production of the wing panels and the fuselage of the Eclipse 500 aircraft, the rear ramp of the Boeing C-17 Globemaster III transport plane, the fuselage panels of the Airbus A380, and the construction of the Boeing 747 Large Cargo Freighter [13]. The use of the FSW method as a substitute for conventional methods of joining using rivets or bolted connections enables the acceleration of the production process thanks to its automation and the elimination of problems related to making holes for fasteners (notch effect) [14,15].

The welding speed, tool rotational speed, tilt angle, axial force exerted by the tool, tilt angle of the tool, tool depth, and tool geometry are the main FSW process parameters influencing temperature distribution, material mixing quality, and material microstructure, which in turn influences the strength of the FSW joint. Characteristic FSW zones have been classified by Weiss [16]. They include the HAZ, weld nugget (WN), hermos-mechanically affected zone (TMAZ), and BM. The formation of the above regions is influenced by the material flow behaviour under the action of a rotating nonconsumable tool [17]. The tool rotational speed determines the rate of heating in the contact area; the friction force affects the heating rate in the HAZ.

The efficient use of the statistical design of experimental techniques allows the development of an empirical methodology in order to incorporate a scientific approach into the welding procedure [18]. Rather than developing analytical or empirical models based on analysis of variance (ANOVA), methods based on Central Composite Design, Response Surface Methodology (RSM), the use of artificial neural networks (ANN) [19], and genetic algorithms (GAs) [20] provide alternative methods for the optimisation of FSW parameters. Elatharasan et al. [21] used RSM to optimise the process parameters for dissimilar aluminium alloys (EN AW-6061-T6 and EN-AW7075-T6) for ultimate tensile strength (UTS). It was found that the UTS of the FSWed joints increases with an increase in the tool rotational speed and welding speed up to a maximum value and then decreases. Dhancholia et al. [22] used RSM to optimise the parameters and responses taken as the mechanical properties, such as tensile strength, yield strength, and impact strength, of dissimilar EN AW-6061 and EN AW-7039 aluminium alloy joints. The desirability method for solving multiple response problems has shown that tool rotational speed and feed rate make a large contribution to producing the necessary frictional heat and have a significant impact on the mechanical properties of the joint. Mohamed et al. [23] optimised the FSW parameters in the joining of dissimilar alloys (EN AW-5083 and EN AW-6061) using Taguchi-based grey relational analysis under different process parameters. ANOVA has shown that the tool rotational speed was the most influential parameter in determining the high strength of joints followed by the axial force and feed rate. Rajakumar et al. [18] used design sensitivity analysis (DSA) and central composite face-centred (CCF) design to optimise the FSW process and tool parameters to attain a maximum tensile strength in EN AW-7075-T6 aluminium alloy joints. They concluded that the tool rotational speed was more sensitive than the other parameters, followed by the welding speed, axial force, and pin diameter. Prasad and Namala [24] used ANOVA to optimise the FSW process of EN-5083 and EN AW-6061 plates of 5 mm thickness. They found that the tunnel defects were found in all joints fabricated with tapered cylindrical tool pin profiles. The most efficient way of designing an experiment can be achieved using the Taguchi method [25,26], which allows one to find the most significant FSW process parameter among the parameter combinations using the signal-to-noise ratio (S/N) [27]. Chandran et al. [28] used the Fisher test to find the design parameter which had the greatest effect on the shear strength characteristic of the Friction Stir Lap Welding of EN AW-6061-T6 aluminium alloy sheets. The results indicated that tool rotational speed had the maximum percentage contribution (51%) on shear strength. The percentage contributions of feed rate and axial load were 38% and 8%, respectively (with a 3% error). Abbas et al. [29] used the design of the experiment to obtain the optimum FSW parameters by utilising the Taguchi technique based on the ultimate tensile test results. The results showed that the optimum friction stir welding parameters were a 0.25 mm shoulder when welding 3 mm-thick EN AW-7075-T6 aluminium alloy joints. Aram and Zemri [30] applied the design of the experiment (DoE) to determine the most important factors that influence the hardness and UTS of EN AW-6082-T6 joints produced by FSW. They successfully used Taguchi’s orthogonal array to find the optimum-level settings of the process parameters. Bora et al. [31] presented a feasibility study of FSW to join Cu-alloy and Al-alloy sheets. The experiment was conducted using a general full factorial design by varying the feed rate, probe offset, and tool rotation speed and by keeping the plunge depth constant. The maximum tensile properties were obtained at a higher tool rotational speed by keeping the welding speed constant. Moreover, the UTS of the FSW joints decreased as the probe offset lessened. Bayazid et al. [32] analysed the effects of welding parameters on the mechanical properties and microstructures of the FSW joints of EN AW-7075 and EN AW-6063 aluminium alloys via ANOVA and the Taguchi method. ANOVA analysis indicated that the effectiveness of the welding speed and tool rotational speed on the tensile strength of the joints were 30% and 59%, respectively. Raweni et al. [33] applied the Taguchi design to find out the optimised set of FSW parameters in terms of total crack propagation and crack initiation energy in weldments. The contributions of three welding parameters (tilt tool angle, welding speed, and tool rotational speed) to the fracture toughness energy in friction stir welding of EN AW-5883 aluminium alloy sheets were successfully determined. Navale and Borkar [34] analysed the UTS of dissimilar Al-Cu plates using the Taguchi orthogonal array. It was found that the tool rotational speed was the most significant factor with a contribution of 70.44%. Nazaelou et al. [35] optimised the FSW parameters with ANOVA and the Taguchi method for maximum electrical conductivity in EN AW-1080-welded sections. They concluded that the main effective parameter for the materials transfer mechanism under the pin was the welding speed. Jain and Kumar [36] conducted a multi-response optimisation of the process parameters in FSWed AW-6061-T6 aluminium alloy sheets using Taguchi grey relational analysis and ANOVA. The microhardness and UTS were used to evaluate weld quality. The ANOVA results indicated that the tool rotational speed was the most significant parameter followed by the tool pin profile and welding speed. Tansel et al. [19] applied a genetically optimised neural network system to optimise the process parameters of friction stir welding of EN AW-1080 aluminium alloy sheets. The optimal operating conditions were estimated using the GA. Mishra [37] focused on the optimisation task for the FSW process in order to obtain the maximum ultimate tensile strengths of EN AW-6061-T6 aluminium alloy joints. The decision trees regression model and ANNs were selected for the purpose. It was concluded that the ANN algorithm gives a better and more accurate result than the decision tree regression algorithm. Shehabeldeen et al. [38] optimised the FSW process using an adaptive neuro-fuzzy inference system integrated with a Harris Hawks Optimizer. The joints were examined for the EN AW-2219-T87 aluminium alloy plate. It was found that the tool rotational speed was the most effective parameter in the UTS. Andersen et al. [39] applied ANN for the first time in welding applications to predict the shape of a welding bead during the gas tungsten arc welding process. ANN was also used to predict the correlation between FSW process parameters and mechanical properties of welded joints [19,40,41]. Aliha et al. [42] analysed the FSW process of dissimilar EN AW-7277-T6 and EN AW-6061-T6 aluminium plates. Using the ANN approach, the UTS and hardness of the tested joints were predicted for different FSW process variables including the pin speed and material position on the retreating side and advanced side. The location of the fracture in the tensile test was shown to be in the areas with minimum hardness values. Okuyucu et al. [43] developed an ANN model for the analysis and simulation of the correlation between the FSW parameters of aluminium joints and the mechanical properties (yield strength, UTS, elongation). It was found that the correlations between the predicted and measured values of the UTS, hardness of BM, and hardness of HAZ were better than those of the yield strength and elongation.

The essence of the research was the selection of FSW parameters for joining stiffening elements (Z-stringers) to a thin-walled structure (skin). Such a structure is used in aircraft; hence, the considerations are focused on the joining of the overlapping sheets of the 2024-T3 alloy, which is commonly used in the aircraft industry. This paper presents the results of the experimental studies. On this basis, a multi-criteria optimisation was carried out, which was designed to indicate the appropriate parameters of the linear FSW process. From the viewpoint of application in the aircraft industry, it is important to optimise the FSW parameters for the maximum mechanical strength of the joint. The vast majority of articles reviewed concern the optimisation of welding parameters for only one selected output parameter (most frequently joint strength). The aim of the multi-criteria optimisation was to determine the most favourable combination of parameters in terms of both the smallest dispersion and highest load capacity of joints. The experimental research was carried out based on a three-factor three-level full factorial Design of Experiments (DoE) plan. The welding speed (feed rate), tool rotational speed, and pin length were considered the parameters in the FSW process.

The scientific novelty presented in this article is the selection of optimal welding parameters ensuring a high load capacity of the joint while maintaining high repeatability. Many authors take up the problem of optimising the parameters of the welding process using the FSW method, usually taking into account only one criterion, which is the strength of the joint. In addition to the strength, the issue of the quality of the joint structure is also discussed in many other works. Of course, when examining a relatively new technology that is still being developed, these are extremely important issues, but from the point of view of the possibility of implementing FSW technology for industrial applications, it is also important to ensure repeatability of the strength of the joints produced as well as the repeatability of the quality of the weld structure. Therefore, an analytical optimisation was carried out in which two criteria were adopted, i.e., both maximising the strength of the joint as well as minimising the standard deviation. The issue raised in the paper originates from the research on the technology of thin-walled aircraft structures; the presented research relates specifically to the skin-stringer joint present in the structures of aircraft fuselages. Hence, the lap joints of materials used in aviation, i.e., aluminium alloy EN AW-2024-T3, were investigated. The high quality of aircraft structures requires high repeatability of the process, hence the considerations presented in the paper are in line with the current technological trends.

## 2. Material and Methods

### 2.1. Material

The subject of the research was the lap joints of 1 mm-thick sheets made of EN AW-2024-T3 aluminium alloy. The chemical composition and basic mechanical properties of the test material are summarised in Table 1 and Table 2, respectively.

### 2.2. Welding Procedure

**Figure 1:**
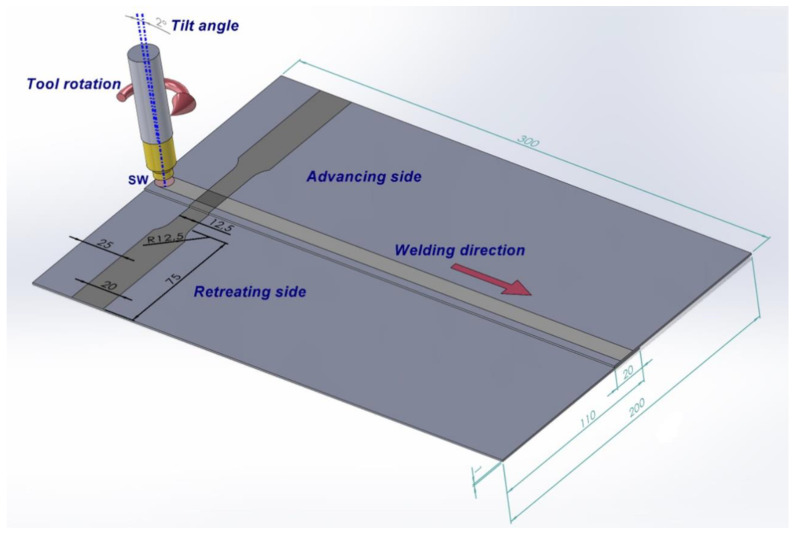
Configuration of the welded panels (unit: mm).

Two sheets with dimensions of 1 mm (thickness) × 100 mm (width) × 300 mm (length) were friction stir welded with an overlap of 20 mm. The welded panels were then cut into 12.5 mm-wide samples with the shape and dimensions shown in Figure 1. The samples were cut using an AL-750SA wire EDM machine supplied by AccuteX (Taichung City, Taiwan). The specimens were cut omitting the initial and final sections of the weld 25 mm in length to minimise the effect of instability in the process during its initiation and completion.

**Figure 2:**
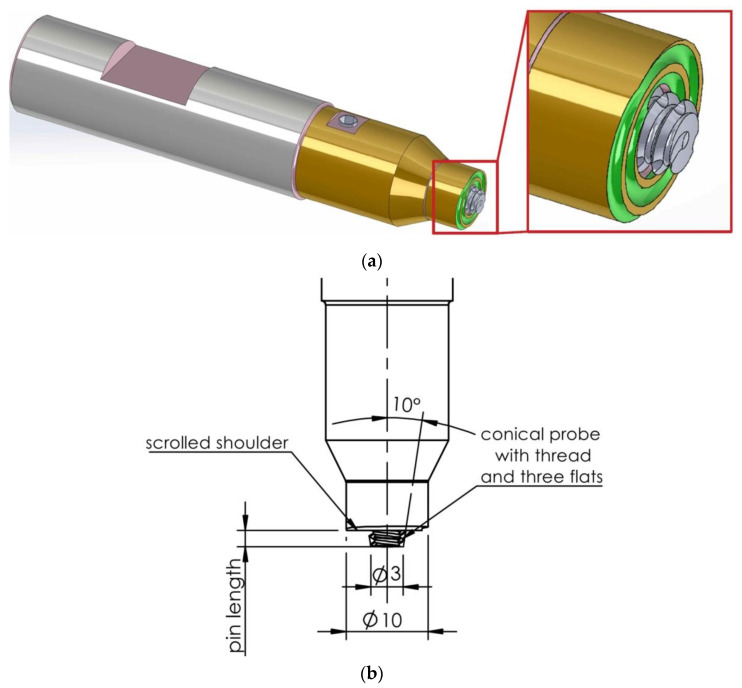
(**a**) View and (**b**) dimensions of the FSW tool (unit: mm).

A 10-K-4-Z-F-B tool supplied by EINSAL East Sp. z o.o. (Mikołów, Poland) with a shoulder diameter of 10 mm and a pin diameter of 3 mm was used for welding. A scrolling feature was used on the front surface of the shoulder. A pin with a parallel flute and an adjustable length in a range between 1.1 and 1.9 mm was used (Figure 2a,b). The welding process was carried out on a 3-axis HAAS TM1P milling machine; position control was used in the research.

**Figure 3:**
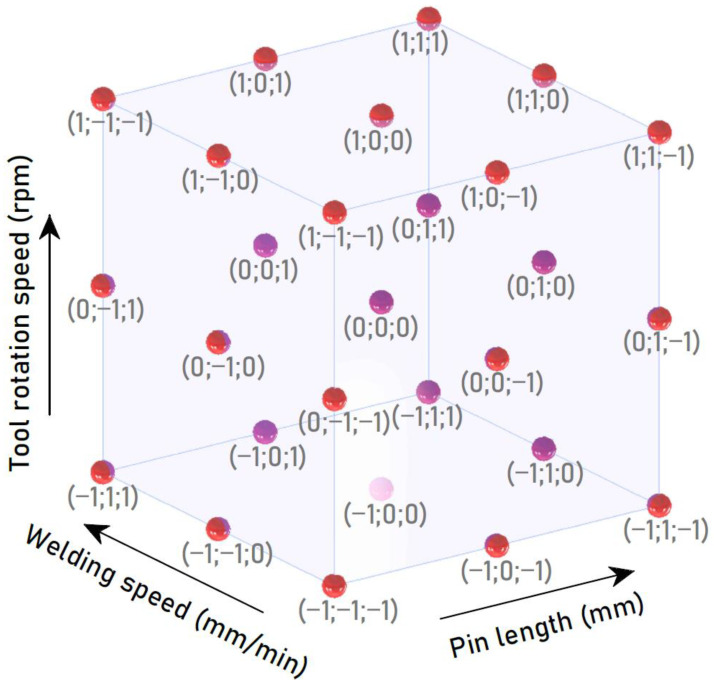
The 3D design space of welding parameters.

Preliminary research identified the boundary values of the welding process parameters on the basis of which the full factorial DoE was created. In accordance with the DoE plan, the values of the welding parameters were adopted on three levels; therefore, 27 combinations of parameters were investigated. The complete research plan as a design space for a 3-factor 3-level full factorial DoE is presented in Figure 3. The range of variability of the values of the welding parameters was determined in preliminary experimental studies. The experimental plan adopted is listed in Table 3. For each variant, one panel was welded from which four specimens were cut; three of the specimens were intended for strength tests (tensile/pure shear test, samples denoted A, B, C) and one specimen was intended for metallographic analysis (sample D).

**Figure 4:**
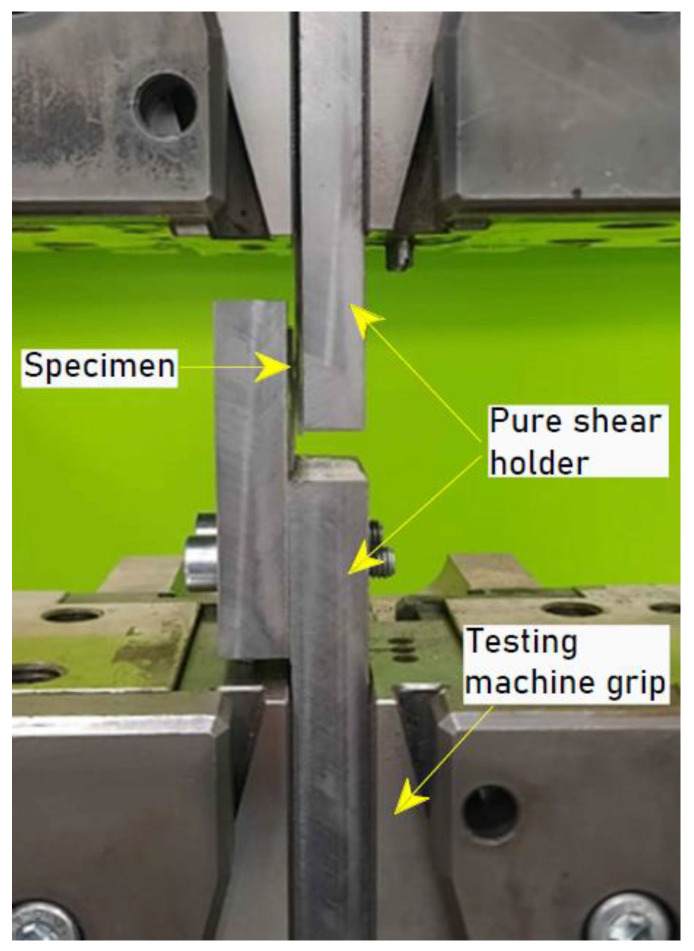
Configuration of the pure shear holder in the grippers of the tensile testing machine.

Due to the complex state of stresses occurring in the static tensile test, which makes it difficult to compare certain variants of welded joints, it was proposed to conduct a static tensile/pure shear test with the use of a grip (Figure 4), which perceives selected degrees of freedom of the specimen, preventing the single-lap joint from bending under the influence of bending moments. Thus, the normal components of stresses are minimised.

Static strength tests using the tensile/pure shear test were carried out in accordance with the recommendations of the ISO 14273 standard [45]. The tests were carried out at room temperature using the Zwick/Roell Z100 (Ulm, Germany) testing machine with a testing speed equal to 2 mm/min. Due to the applied pure shear device, the strength results obtained for the individual variants were compared to the tensile strength of the base material, determining the relative tensile strength. When determining this value, the load capacity of the joints was related to the cross-sectional area of the sheet in tension in the area of the base material, i.e., with dimensions of 12.5 mm × 1 mm.

### 2.3. Optimisation of the Welding Parameters

In aircraft construction, apart from the strength that determines the quality of the joint, an equally important parameter is the standard deviation of the results obtained. A lower value of deviation is related to the greater repeatability of the process, which is reflected in the reliability of the structure. Based on the results of experimental investigations, analytical optimisation was carried out to indicate the most favourable combination of parameters in terms of both the smallest dispersion and highest load capacity.

The standard deviation of the test results was determined based on 5-element samples. Evaluation of the repeatability (stability) of the FSW process was performed using the Hartley test. In order to verify the hypothesis on the repeatability of variance, the Fmax statistic of the test was determined:

(1) $$F_{\max}=\frac{S^{2}{\left(y\right)}_{I\;\mathrm{maz}}}{S^{2}I}$$

where $S^{2}{\left(y\right)}_{i\;\min}$ and $S^{2}{\left(y\right)}_{i\;\mathrm{maz}}$ are the minimum and maximum values of variance from the set of all variances. The critical values of the Hartley test, f_max(α,k,v)_ for the number of degrees of freedom k = m = 27 and v = r − 1 = 3 − 1 = 2 and for the significance level α = 0.05, were read from statistical tables and compared with the calculated value of F_max_.

The optimisation analyses were carried out for all combinations of the welding parameters, the values of which varied within a wide range (Table 3). For the purpose of the optimisation analysis, the following designations of the welding parameters were adopted: x_1_—pin length, x_2_—feed rate, x_3_—spindle rotational speed.

The presented method of the analytical multi-criteria optimisation proves the originality of this work, which enables the indication of the parameters of the FSW process, which will ensure not only the high strength of the joint but also the repeatability of the process. In addition to the optimisation criteria, which are the values of the load capacity of the joints as well as the values of the standard deviation, metallographic analyses were also performed to discuss the features of the structure of the joints made with the selected parameters. The implementation of fractographic analyses made it possible to expand the discussion on the influence of the parameters on the mechanism of the joint failure.

## 3. Results and Discussion

### 3.1. Tensile/Pure Shear Tests

**Figure 5:**
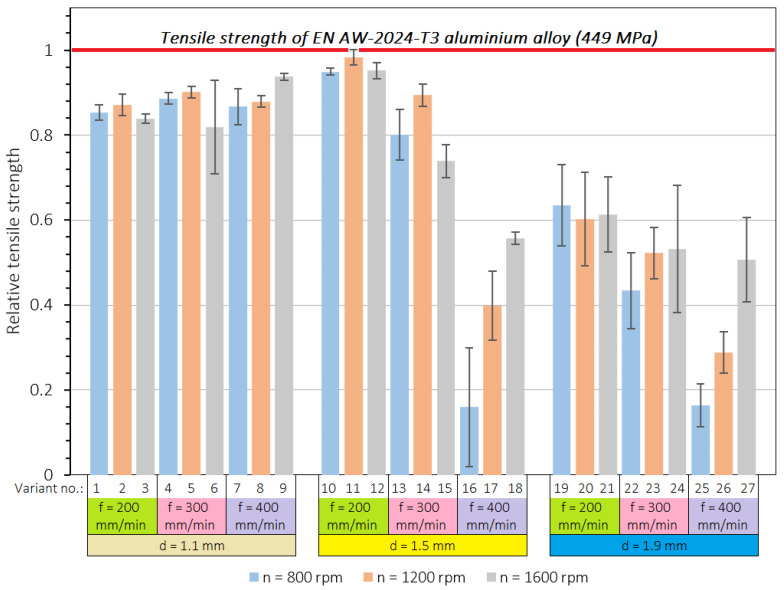
Effect of welding parameters on relative tensile strength.

The quantitative results of the tests in the form of the value of the maximum tensile forces for each of the samples are summarised in Table 4, including the mean value for each variant of the input parameters, as well as the standard deviation (SD). The cumulative diagram (Figure 5) presents the relative tensile strength for individual variants with respect to the tensile strength of the base material. The highest load capacity of the joint was obtained for the following combination of parameters: pin length 1.5 mm, welding speed 200 mm/min, and tool rotational speed 1200 rpm. In this case, the mean value of the load capacity was 5.527 kN and a relatively low scatter of the results was obtained (SD = 0.103 kN). In this case, the tensile failure mode was used; therefore, the tensile strength was determined as 442.16 Mpa, which was 98.5% of the strength of the base material. A slightly lower value of the load capacity was also shown for the same values of pin length and welding speed but the other values of tool rotational speed. For a tool rotational speed of 800 rpm, the mean value of the load capacity was 5.338 kN (SD = 0.043 kN), whereas at a tool rotational speed of 1600 rpm, the load capacity was 5.352 kN (SD = 0.102 kN). The increase in the welding speed at the chosen value of the pin length caused a decrease in the load capacity of the joint as well as a significant increase in the dispersion of the results. Among the combinations of parameters that were considered, the lowest load capacity was found in joints made using the following parameters: pin length 1.5 mm, welding speed 400 mm/min, and tool rotational speed 800 rpm. In this case, the mean value of the load capacity was 898 N and the significantly greatest dispersion of the results (SD = 0.127 kN) was also noted.

**Figure 6:**
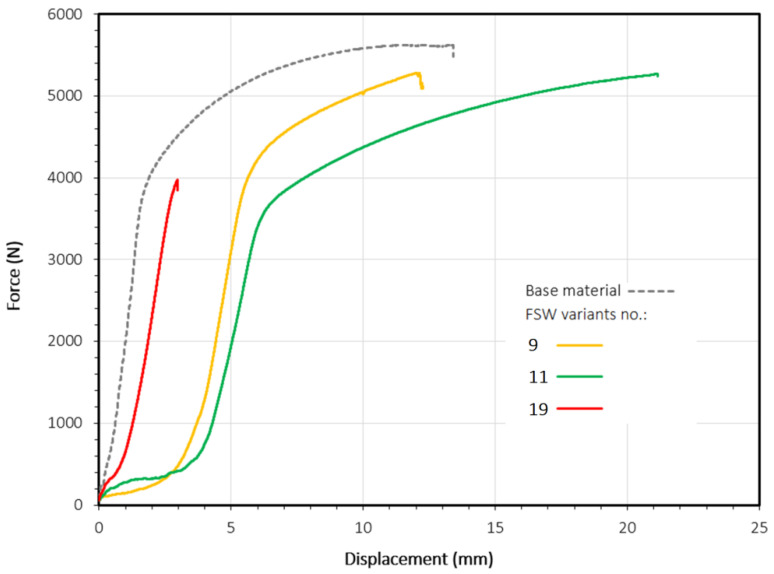
Force–displacement curves determined in the tensile/pure shear tests for FSW variants no. 9, 11, and 19.

In the case of pin length 1.5 mm, the welding process lost its stability with an increase in welding speed, because a welding speed of 400 mm/min resulted in a significant decrease in joint strength and a significant lack of repeatability. The smallest of the values of pin length evaluated, 1.1 mm, ensured the most stable welding conditions because, regardless of the other parameters, the average values of the load capacity of joints were kept in a relatively narrow range. In turn, the highest pin length value of 1.9 mm did not provide adequate strength in any combination of input parameters; these results oscillated in the restricted range between 18 and 64% of the tensile strength of the base material. Figure 6 shows the force–displacement curves for the most resistant joints obtained at a tool rotational speed of 800 rpm (variant no. 9), 1200 rpm (variant no. 11), and 1600 rpm (variant no. 19).

Generally, with pin lengths equal to 1.5 mm and 1.9 mm, significant dispersions of the results of the load capacity of the joints were observed. With a larger pin length, a larger volume of material must be plasticised in order to obtain an effective joint. It was observed that an increase in the welding speed led to a decrease in the load capacity here and low repeatability was also noticed in these areas. Thus, it can be seen that too high a welding speed did not ensure stable process conditions; there was a lack of sufficient plasticisation of the material, which resulted in the formation of various types of structural defects and a lack of repeatability and effective formation of the joint structure.

**Figure 7:**
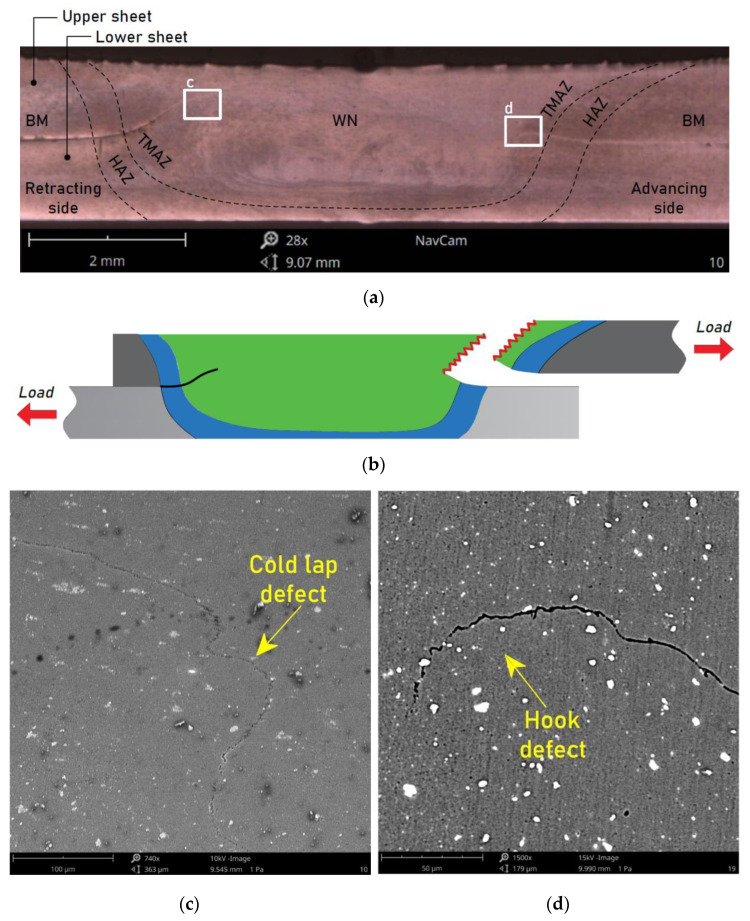
(**a**) Cross-sectional view of the FSW sample, (**b**) failure mode, and (**c**,**d**) magnification of the areas c and d, respectively, for a weld made with the parameters d = 1.9 mm, f = 400 mm/min, and n = 800 rpm.

Figure 7a shows a cross-section of the weld that was made using the following combination of parameters: pin length 1.9 mm, welding speed 200 mm/min, and tool rotational speed 800 rpm. The zones typical for FSW joints, i.e., the weld zone, thermo-mechanically affected zone, and HAZ, were distinguished. The weld consisted of the WN and the TMAZ. The samples in the tensile/pure shear test repeatedly failed due to stretching of the upper sheet in the TMAZ area from the advancing side. This was a result of the modified microstructure of the material in relation to the base material. The base material in the vicinity of the TMAZ was transformed during the welding process as a result of the impact of the heat and significant plastic deformation, which led to a weakening of the material. In addition, microscopic observations using SEM showed that there was no metallic connection in the hook defect area (Figure 7d) and this defect was located above the interface of the sheets, which resulted in a reduction of the upper sheet cross-section, which in turn resulted in a relatively low load capacity of the joint and the specific tensile fracture mode of joint destruction (Figure 7b). The metallographic analysis also made it possible to identify a typical defect for FSW joints, namely a cold lap defect on the retracting side of the weld (Figure 7c). As in the case of the hook defect, there was no metallic continuity in the cold lap area. The uniformity of the microstructure was influenced by the tool geometry (size of shoulder and pin) [17]. The tool geometry also played a critical role in the flow of the plasticised material and localised heating. The stirring and movement of the material were affected by the design features of the tool pin profile. So, from the heating aspect, the relative size of the pin and shoulder is important. The workpiece material below the shoulder should be sufficiently stirred and moved towards the trailing side [17].

**Figure 8:**
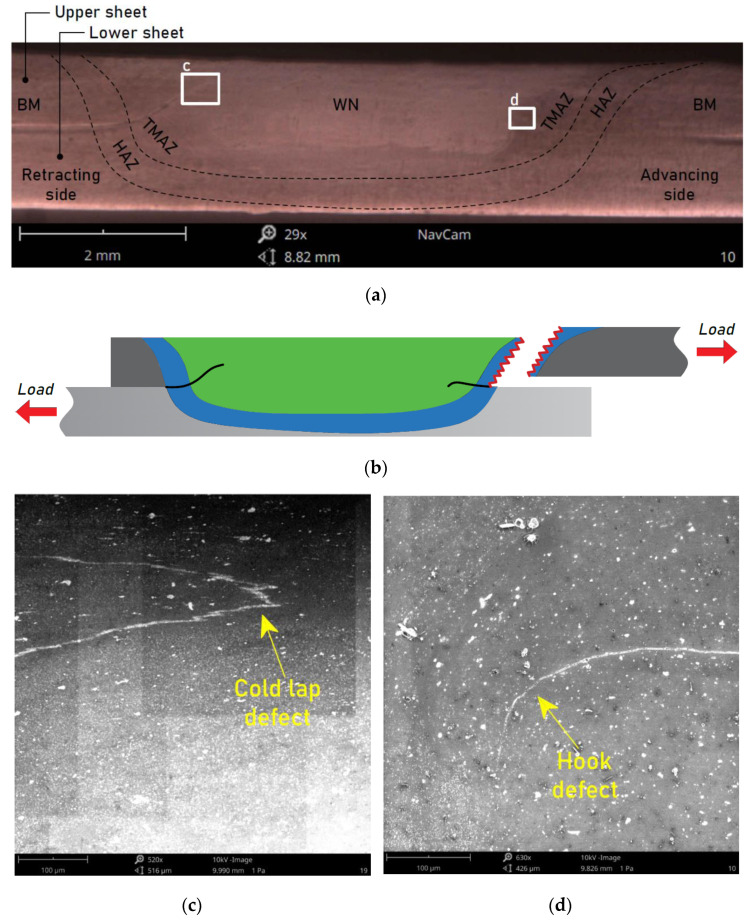
(**a**) Cross-sectional view of the FSW sample, (**b**) failure mode, and (**c**,**d**) magnification of the areas c and d, respectively, for a weld made with the parameters d = 1.5 mm, f = 200 mm/min, and n = 1200 rpm.

FSWed joints made at pin length 1.5 mm, welding speed 200 mm/min, and various values of tool rotational speed showed the highest load capacity of the joint. Figure 8a shows a macroscopic view of the cross-section of the weld made with the welding parameters: pin length d = 1.5 mm, welding speed 200 mm/min, and tool rotational speed 1200 rpm. As in the case previously described, the joint characteristics are also listed here. On the macroscopic scale, no significant differences were noted between the variants except for the areas of occurrence of individual phases, which resulted from the difference in the pin length. At the microscopic scale, it was observed that despite the occurrence of defects such as cold lap (Figure 8c) and hook (Figure 8d), in this case, a metallic connection between sheets took place, thanks to which the continuity of the material was maintained, and therefore the weakening of the sheets in the upper sheet (Figure 7b) did not occur. The failure was also due to the stretching of the upper sheet but in the HAZ (Figure 8b).

**Figure 9:**
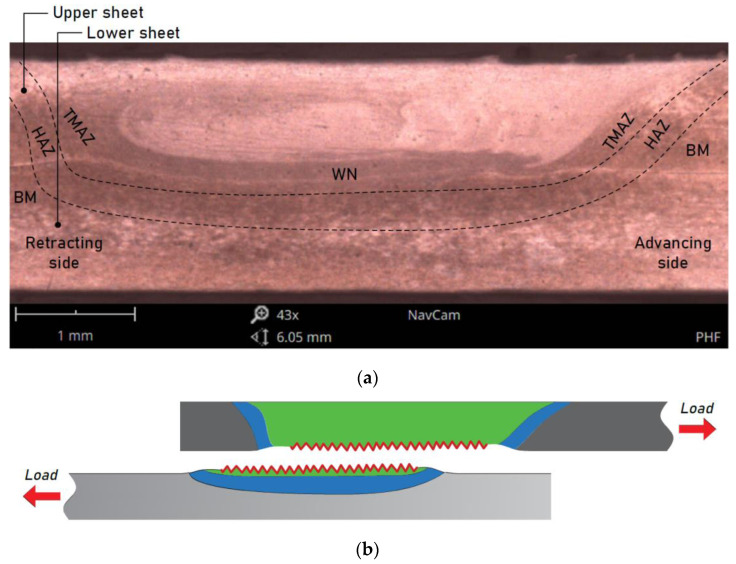
(**a**) Cross-sectional view of the FSW sample and (**b**) shear failure mode for a weld made with the parameters d = 1.1 mm, f = 400 mm/min, and n = 1600 rpm.

Now, consider the joints made using the smallest pin length analysed. Figure 9a shows the cross-section of the joint made with the parameters: pin length 1.1 mm, welding speed 400 mm/min, and tool rotational speed 1600 rpm. In this case, the weld nugget had a depth similar to the thickness of the upper sheet. So, the lower surface of the upper sheet almost coincided with the interface between the plates. In this case, the microscopic analysis did not show the above-described cold lap and hook defects. In the case of such FSW joints, a different character of failure was noted, i.e., they were sheared in the plane of the interface (Figure 9b).

**Figure 10:**
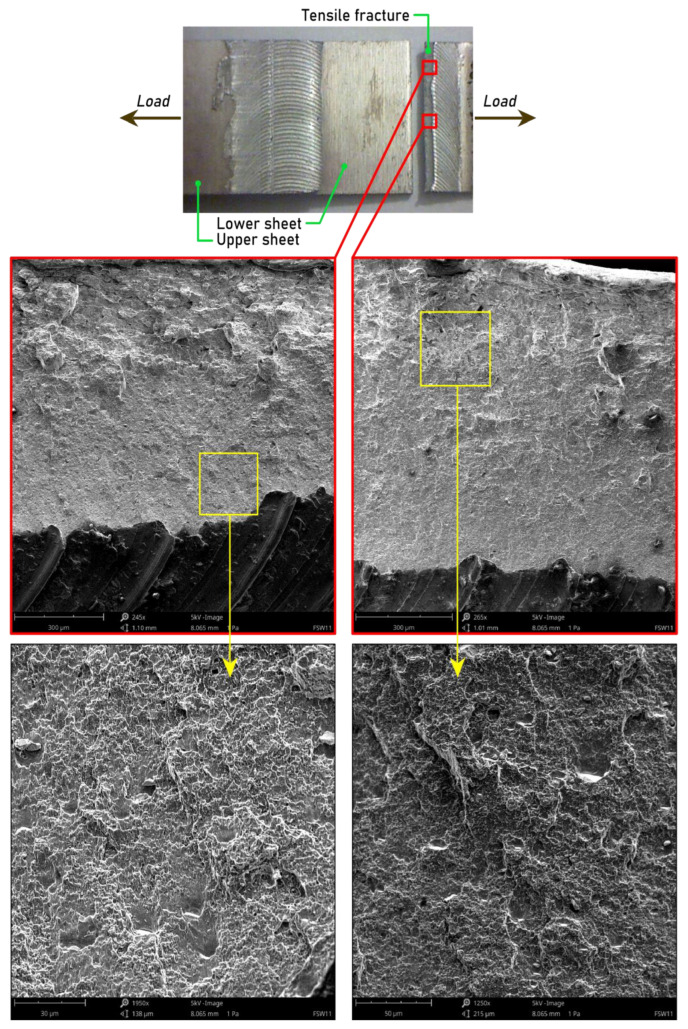
SEM images of a fracture surface of an FSW weld joint made using the following parameters: pin length d = 1.5 mm, welding speed f = 200 mm/min, and tool rotational speed n = 1200 rpm.

**Figure 11:**
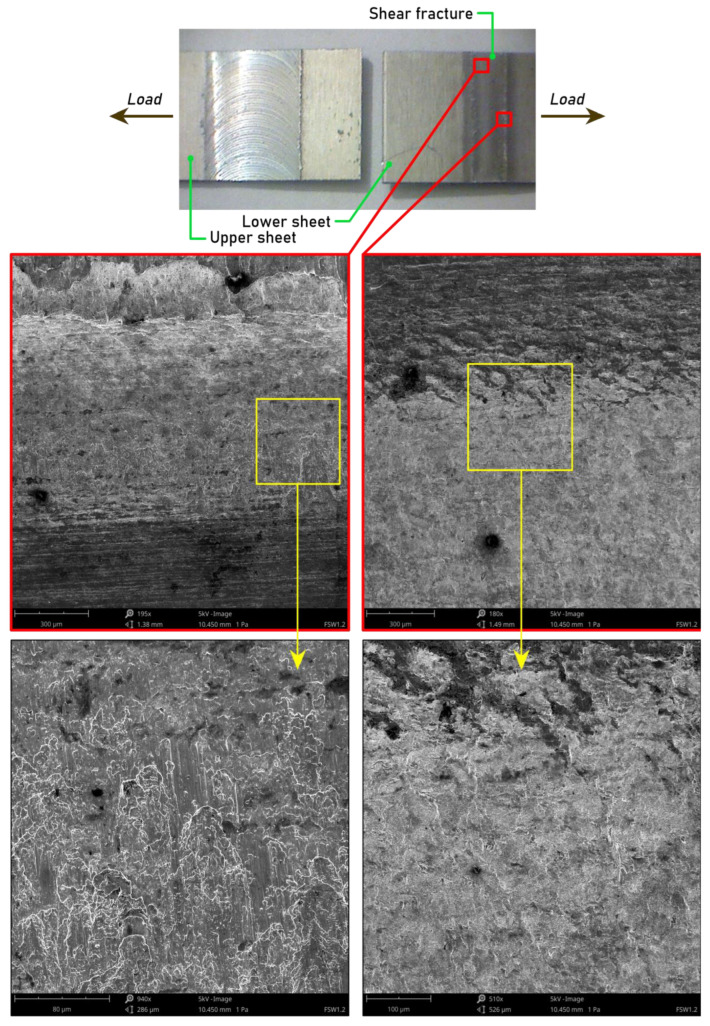
SEM images of a fracture surface of an FSW weld joint made using the following parameters: pin length d = 1.1 mm, welding speed f = 400 mm/min, and tool rotational speed n = 1600 rpm.

**Figure 12:**
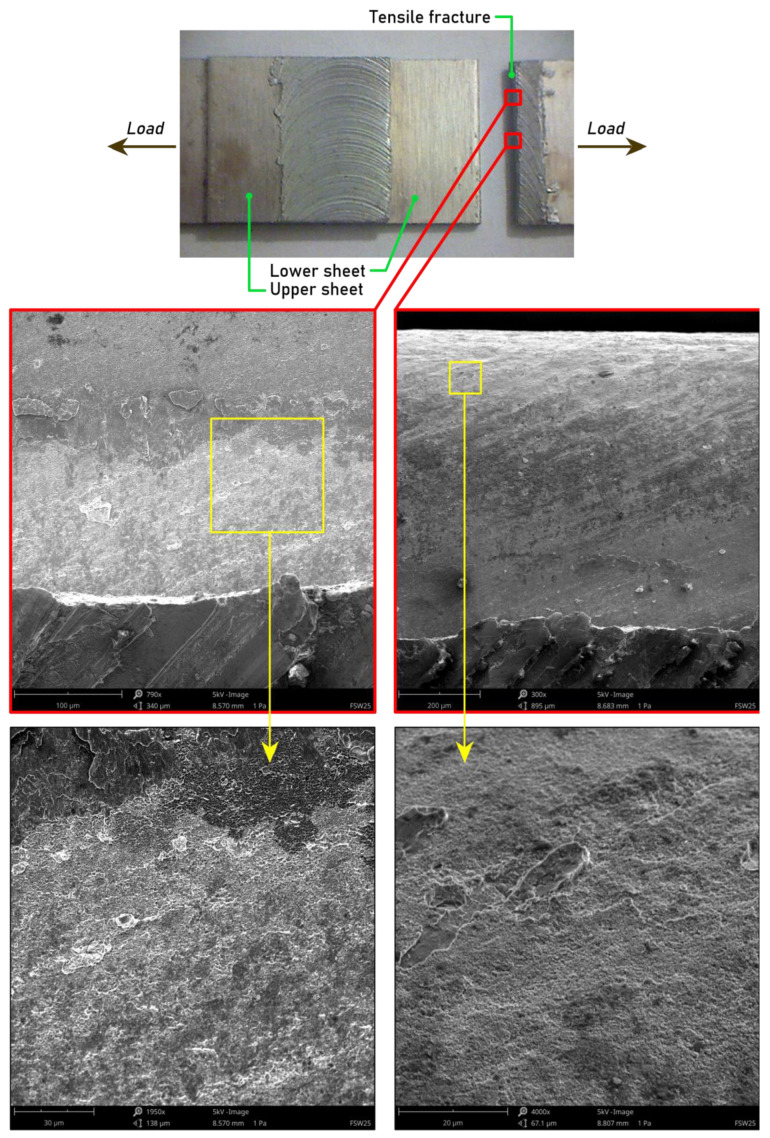
SEM images of a fracture surface of an FSW weld joint made using the following parameters: pin length d = 1.9 mm, welding speed f = 400 mm/min, and tool rotational speed n = 800 rpm.

Figure 10, Figure 11 and Figure 12 show images of the fracture surfaces of specimens no. 9, 11, and 19 corresponding to the force–displacement curve shown in Figure 6. The fracture area of the upper sheet of the joint was made with the following conditions: pin length 1.5 mm, welding speed 200 mm/min, and tool rotational speed 1200 rpm and consisted of two distinct areas of different sizes of dimples that indicated the ductile type of fracture (Figure 10). The size of the dimples depended on their position on the cross-section of the fractured layer. The presence of depressions on the fracture surface resulted from the intercrystalline character of the cracking caused by the nucleation of the microvoids [46].

The crack occurred on the advancing side. During the welding of the lap joints, an asymmetrical transition was created in the advancing side between the BM and the HAZ [47]. It was manifested by the curvature of the contact line between the heat-affected zone and the hook defect. Fracture initiation occurred near the interface between the sheets in the upper sheet. The results of the research carried out by Kudła et al. [47] show a close relationship between the so-called hook line and the weakening in the nugget zone on the advancing side. Crack initiation and propagation took place at the contact line between the joined materials and the weld and its course was consistent with the shape of the end of this line. Tensile fracture mode was also created when stretching sample no. 19 (Figure 12). The thin waves that arose as a result of mixing materials at the interface were intermetallic layers formed between the two joined materials [46]. There was a clear interface separating zones of different-sized dimples. The dimples that lay across the upper sheet showed a similar character, with no visible shearing of the bands, as can be seen in Figure 10.

The most resistant joint made with pin length d = 1.1 mm exhibited shear fracture along the lap interface (Figure 11). When this type of fracture mode occurs, the crack is prone to initiate and propagate from the lap interface [48]. In the joint welded with pin length 1.1 mm, welding speed 400 mm/min, and tool rotational speed 1600 rpm, the upper stir zone and the bottom stir zone had relatively higher strength and there was no prior crack initiation in the joint. Therefore, the fracture simultaneously propagated along the boundaries of the overlaps.

### 3.2. Multi-Objective Optimisation of FSW Parameters

**Figure 13:**
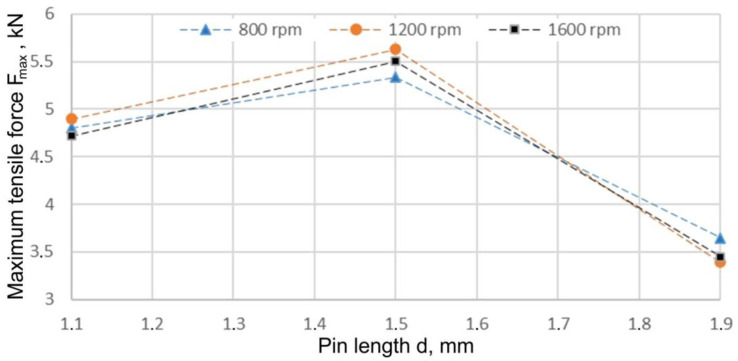
Effect of pin length and tool rotational speed on maximum force transmitted by the joint F_max_ (x_2_ = 200 mm/min).

The first stage of the research was focused on the analysis of the influence of the pin length x_1_ on the load capacity of the joint. For this purpose, welds were made with pin lengths 1.1, 1.5, and 1.9 mm and with three different tool rotational speeds 800, 1200, and 1600 rpm. From the graph showing the dependence of the maximum force borne by the joint F_max_ and the pin length (Figure 13), it was noted that the curves assigned to the various tool rotational speeds showed the same trend. Regardless of the tool rotational speed adopted, the highest strength of the joint was observed for the pin length x_1_ = 1.5 mm. After this value was exceeded, all the characteristics showed a trend of decreasing joint strength with increasing pin length. In the case of the curve corresponding to a tool rotational speed of 1200 rpm, an initial significant increase in the strength of the weld was recorded, reaching the maximum value of 5.62 kN for the pin length of 1.5 mm. Then, with an increase in pin length, the trend reversed and the strength of the joint decreased. In the lap-welded joints, there was an optimal pin length, which guaranteed the right conditions for the plasticisation of the material. Due to the large tool cavity (d = 1.9 mm) close to the sum of the thicknesses of the two sheets (2 × 1 mm), in certain areas of the TMAZ there were no proper conditions necessary to produce a complete metallic joint. Under such conditions, a cold lap defect with an intense outline was observed in the cross-section of the joint. The increase in the plunge depth increased the frictional heat, consequently leading to better material flow, proper mixing, and joint consolidations [49]. At high plunge depths, the nugget zone temperature and the associated heat input increased, which caused grain growth in a dynamically recrystallised fine-grained nugget zone [50]. As a result of high temperatures, the maximum tensile stress decreased due to grain coarsening and local thinning [51].

**Figure 14:**
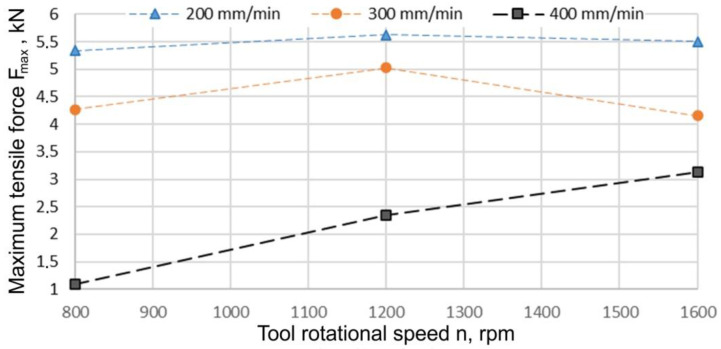
Effect of the tool rotational speed and welding speed on the maximum force transmitted by the joint F_max_ (x_1_ = 1.5 mm).

The increase in the welding speed when the pin length was x_1_ = 1.5 mm resulted in a reduction in the load capacity of the joint (Figure 14). At 1200 rpm, this decrease was equal to 10.67% after increasing the welding speed to 300 mm/min. A further increase in the welding speed (400 mm/min) caused a decrease in the load capacity of the joint by 53.58% to a value of 2.34 kN. In the case of welding speeds of 200 mm/min and 300 mm/min, an increase in the tool rotational speed from 800 rpm to 1200 rpm caused an increase in the load capacity of the joint and then its decrease at a tool rotational speed of 1600 rpm. At a welding speed of 400 mm/min, the load capacity of the joint increased with increasing tool rotational speed. A high tool rotational speed could raise the strain rate, and thereby influence the recrystallisation process, which in turn could influence the UTS [52]. The higher tool rotational speed resulted in a higher temperature and caused the excessive release of stirred materials to the upper surface. In contrast, lower tool rotational speeds resulted in tunnel defects in the weld zone [52,53].

**Figure 15:**
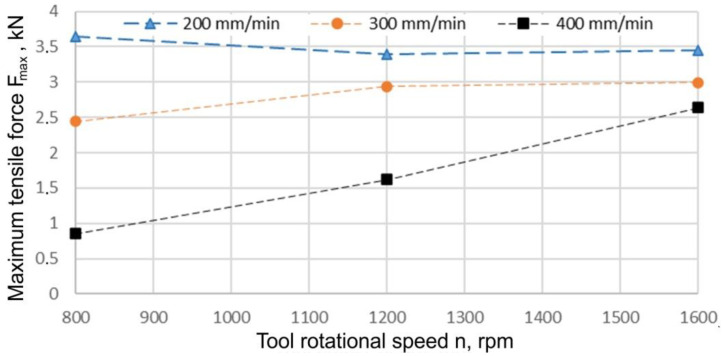
Effect of the tool rotational speed and welding speed on the maximum force transmitted by the joint F_max_ (x_1_ = 1.9 mm).

An increase in the tool length to 1.9 mm caused a decrease in the load capacity of the joint regardless of the value of the welding speed adopted (Figure 15). In the case of a welding speed of 200 mm/min, the highest value of load capacity (3.64 kN) was recorded at a tool rotational speed of 800 rpm. In this case, a further increase in the tool rotational speed did not cause any significant changes in the load capacity of the joint. In the case of a welding speed of 300 mm/min with a tool rotational speed increase from 800 rpm to 1200 rpm, an increase in the load capacity of the joint by 20.57% was observed. A further increase in the welding speed did not cause any significant changes in the strength of the joint. At a welding speed of 400 mm/min, in a similar manner to the pin length of 1.5 mm, a continuous increase in the load capacity of the joint was observed with an increase in the tool rotational speed. So, both the tool rotational speed and welding speed must be optimised to obtain the weld region with particles uniformly distributed throughout the matrix [54]. Elangovan et al. [17] concluded that the maximum temperatures were nearly the same at all rotational speeds. They explained that there were two reasons for this. First, the coefficient of friction decreased when a local melt occurred. Second, the latent heat absorbed some heat input.

**Figure 16:**
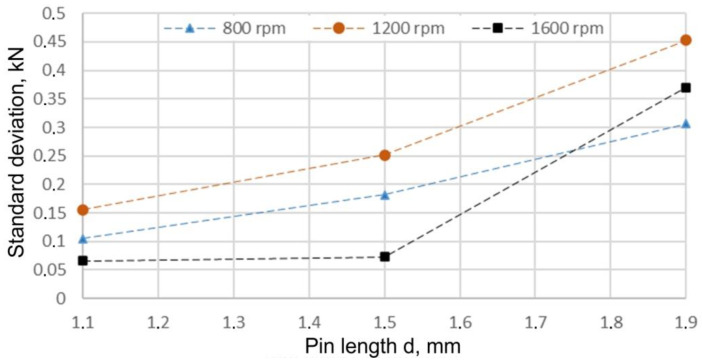
Standard deviation of the test results for a welding speed of x_2_ = 200 mm/min.

The test analysis showed that the results obtained with different values of the input parameters did not have the same variance (F_max_ > f_max(α,k,v)_). The analysis of the test results also showed some dependencies regarding the value of the variance and standard deviation. At a feed rate of 200 mm/min regardless of the value of the tool rotational speed adopted, the standard deviation increased with the increase in pin length (Figure 16). Increasing the rotational speed from 800 rpm to 1200 rpm caused an initial increase in the standard deviation value and then its decrease at a welding speed of 1600 rpm.

The selection of the optimal parameters of the FSW process requires the determination of an adequate mathematical model in the form of a regression function W(x). The regression analysis was carried out using the principle of least squares of the following form of the criterion assessing the quality of approximation:

(2) $$\min R=\min\overset{N}{\underset{i-0}{\sum }}{\left[f\left(x_{i}\right)-W\left(x_{i}\right)\right]}^{2}$$

where the value of the R-function is a measure of the deviation of the approximating function W(x) from the approximated function f(x), i = 1,…, N—number of experiments.

To describe the process, the form of an algebraic polynomial of degree m, containing interactions between the parameters of the FSW process is used:

(3) $$\begin{array}{c} W\left(x\right)=b_{0}+\overset{S}{\underset{i=1}{\sum }}b_{i}^{\left(1\right)}x_{i}\\ \;\;\;\;\;\;\;\;\;\;\;\;\;\;\;\;\;\;\;\;\;\;\;\;\;\;\;\;\;\;\;\;\;+\overset{S}{\underset{\begin{array}{c} i,\;j=1\\ i<j \end{array}}{\sum }}b_{\mathrm{ij}}^{\left(1\right)}x_{i}x_{j}+\overset{S}{\underset{\begin{array}{c} i,\;j,\ldots ,\;l,n=1\\ i<j,\ldots ,l<n \end{array}}{\sum }}b_{\mathrm{ij}\ldots \ln}^{\left(1\right)}x_{i}x_{j}\ldots +x_{l}x_{n}\\ \;\;\;\;\;+\overset{S}{\underset{\begin{array}{c} i,\;j=1\\ i\neq j \end{array}}{\sum }}b_{\mathrm{ij}}^{\left(2\right)}x_{i}^{2}x_{j}+\overset{S}{\underset{i-1}{\sum }}b_{\mathrm{ii}\ldots m}^{\left(m\right)}x_{i}^{m} \end{array}$$

where there are L unknown coefficients $b_{0},{\;b}_{i}^{\left(1\right)},{\;b}_{\mathrm{ij}}^{\left(1\right)},{\;b}_{\mathrm{ij}\ldots \ln}^{\left(1\right)},{\;b}_{\mathrm{ij}}^{\left(2\right)},{\;b}_{\mathrm{ii}\ldots m}^{\left(m\right)},\;i,\;j,\;\ldots ,\;n=1,\;\ldots ,\;S$ (number of variables in the polynomial (3)), i < j < … < l < n.

The significance of the coefficients of the regression Equation (3) was assessed by comparing their values with the critical value determined from the formula

(4) $$b_{\mathrm{kr}}=t_{\left(\alpha ,\;f\right)}\sqrt{\frac{S^{2}\left(y\right)}{\mathrm{Nr}}}$$

where $t_{\left(\alpha ,\;f\right)}=t_{\mathrm{kr}}$ is the test value of the t coefficient determined from the Student’s t distribution tables, r is the number of repetitions, and N is the total number of experiments.

The Fisher–Snedecor test was used to assess the adequacy of the regression Equation (3) with the research results. At the first stage of the analysis, the adequacy variance was determined according to the formula

(5) $$S_{\mathrm{ad}}^{2}=\frac{r\sum_{i=1}^{N}{\left(\bar{y_{i}}-\overset{=}{y_{i}}\right)}^{2}}{N-k-1}$$

where ${\bar{y}}_{i}$ is the mean value of the measurement results in the i-th experiment, $\overset{=}{y_{i}}$ is the value calculated from the regression equation for the levels of the input and output factors of the i-th experiment, and k is the number of regression equation terms (without the intercept) after excluding the irrelevant terms.

Then, the value of the test coefficient *F* determined according to the formula

(6) $$F=\frac{S_{\mathrm{ad}}^{2}\left(y\right)}{S^{2}\left(y\right)}$$

was compared with the critical value determined from the Fisher–Snedecor distribution tables, obtaining an adequate regression equation W_F_(x) describing the effect of the FSW parameters on the load capacity of the joint in the following form:

(7) $$\begin{array}{c} W_{F}\left(x\right)=b_{0}+b_{1}^{\left(1\right)}x_{1}+b_{2}^{\left(1\right)}x_{2}+b_{3}^{\left(1\right)}x_{3}+b_{12}^{\left(1\right)}x_{1}x_{2}\\ \;\;\;\;\;\;\;\;\;\;\;\;\;\;\;\;\;\;\;\;\;\;\;\;\;+b_{13}^{\left(1\right)}x_{1}x_{3}+b_{23}^{\left(1\right)}x_{2}x_{3}+b_{12}^{\left(2\right)}x_{1}^{2}x_{2}\\ \;\;\;\;\;\;\;\;\;\;\;\;\;\;\;\;\;\;\;\;\;\;\;\;\;+b_{21}^{\left(2\right)}x_{2}^{2}x_{1}+b_{13}^{\left(2\right)}x_{1}^{2}x_{3}+b_{23}^{\left(2\right)}x_{2}^{2}x_{3}\\ \;\;\;\;\;\;\;\;\;\;\;\;\;\;\;\;\;\;\;\;\;\;\;\;\;\;\;\;\;+b_{11}^{\left(2\right)}x_{1}^{2}+b_{22}^{\left(2\right)}x_{2}^{2}+b_{33}^{\left(2\right)}x_{3}^{2}+b_{111}^{\left(3\right)}x_{1}^{3}\\ \;\;\;\;\;\;\;+b_{222}^{\left(3\right)}x_{2}^{3}+b_{333}^{\left(3\right)}x_{3}^{3} \end{array}$$

The values of the coefficients in the regression function describing the load capacity of the joints W_F_(x) and the standard deviation of the scatter of the results W_σ_(x) is given in Table 5.

**Figure 17:**
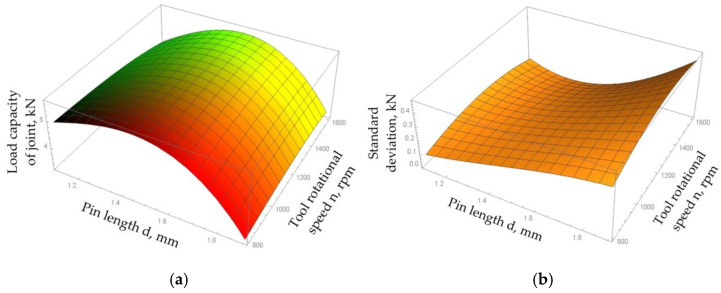
Plots of the regression function of (**a**) load capacity of the joint and (**b**) standard deviation of the process spread for a welding speed x_2_ = 200 mm/min.

In the case of the regression function describing the load capacity of the joint, the mean square error between the results obtained as a result of tests and mathematical modelling was 4.05%. This function reached a maximum value of 5.71 kN at a pin length of 1.48 mm, welding speed of 200 mm/min, and tool rotational speed of 1303 rpm. Figure 17a shows the effects of the tool rotational speed and pin length on the load capacity of the joint determined using the regression function for a welding speed of 200 mm/min. The graph shows the increase in the load capacity of the joint with the increase in pin length. The local maximum of the function is exhibited for a pin depth of 1.48 mm. At lower values of tool rotational speed, the standard deviation of the process increased with the increase in pin length. In the case of higher values of tool rotational speed, the regression function describing the standard deviation of the load capacity of the joint reached a local minimum and then its value rapidly increased (Figure 17b).

**Figure 18:**
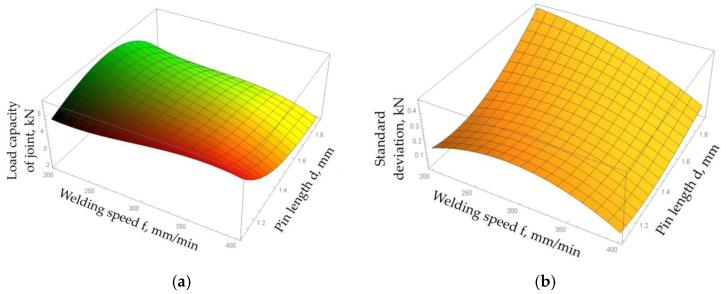
Plots of the regression function of (**a**) load capacity of the joint and (**b**) standard deviation of the process spread for a tool rotational speed x_2_ = 1303 rpm.

The analysis of the graph of the regression function of the load capacity of joints obtained for a rotational speed of 1303 rpm showed that the load capacity of the joint decreased with the increase in welding speed (Figure 18a). The value of the standard deviation of the process spread also decreased with the welding speed (Figure 18b). The local maximum of the regression function of the load capacity (5.71 kN) in a saddle point was found for a welding speed of 200 mm/min. In this case, both increasing and decreasing the pin length caused a decrease in the load capacity of the joint. In addition, an increase in the pin length caused a sharp increase in the standard deviation that reduced the stability of the FSW process. With a welding speed of 400 mm/min, the highest load capacity of the joint was obtained with a pin length of 1.1 mm. In this case, the increase in pin length resulted in a reduction in the load capacity of the joint.

### 3.3. Optimisation of the FSW Process

It is necessary to select the optimal parameters of the FSW process in order to replace conventional methods of joining thin-walled aircraft elements. This selection must guarantee not only the high load capacity of the joints but also the high stability of the process, represented by the lowest possible value of the variance of the results obtained. It requires carrying out a multi-criteria optimisation of the FSW process and finding a compromise solution that meets the conditions presented.

In order to define the multi-criteria problem, the following parameters were adopted:

- D ⊂ R^m^—a set of feasible solutions—the range of the FSW process parameters.
- z = (z_1_, z_2_, …, z_m_) ∈D—an acceptable solution.
- f_i_: D → R—i-th objective function (I = 1, 2, …, k).
- (z) = (f_1_(z), f_2_(z))—objective function of a multi-criteria problem.

The problem of the multi-criteria optimisation of the selection of the FSW process parameters can be written in the form

(8) $$\{\begin{array}{c} f_{1}\left(z\right)=W_{F}\left(x\right)\to \max,\\ f_{2}\left(z\right)=W_{\sigma }\left(x\right)\to \max,\\ z\in D\; \end{array}$$

A one-criteria issue written as

(9) $$f_{1}(z)\;\to \;\mathrm{extremum},\;z\;\in D$$

is the i-th partial issue, whereas the vector $z^{\mathrm{io}}\in D$ in which the i-th objective function achieves the extremum is the i-th partial solution.

Vector

(10) $$\varphi^{0}=\left(f_{1}\left(z^{1o}\right)\right),\;\left(f_{2}\left(z^{2o}\right)\right)$$

is a vector called the ideal (utopian) solution in the evaluation space, whereas:

(11) $$z^{o}=\left(z^{1o},z^{2o}\right)$$

is the solution to the ideal problem. Usually, the solution to the problem (Equation (8)) is not achievable, which means that in the set of feasible solutions D there is no vector z^o^ for which all objective functions achieve the desired extremum. Therefore, while solving the problem presented, effective solutions were sought.

A set of effective solutions usually includes many solutions. Therefore, the aim of the issue that was presented was to select one solution from the set of effective solutions, known as a compromise optimal solution. For this purpose, the problem was reduced to a single-criteria form $s:R^{k}\to R$:

(12) $$\min(F\left(z\right)=\rho \left(\phi \left(z\right),\phi^{o}\right)):z\in D)$$

The regression function describing the load capacity of the joint reached a maximum value of 5.71 kN with a pin length of 1.48 mm, welding speed of x_2_ = 200 mm/min, and tool rotational speed of x_3_ = 1303 rpm, whereas the regression function of standard deviation reached a minimum value of 0.031 kN with a pin length of 1.35 mm, welding speed of x_2_ = 400 mm/min, and tool rotational speed of x_3_ = 1308 rpm. These are partial solutions to the problem described by Equation (8). The ideal (utopian) solution in the space of criteria takes the form:

φ^o^= (f_1_(z^1o^), f_2_ (z^2o^)) = (5.71 kN, 0.031 kN) (13)

The Euclidean distance was adopted as the distance ρ between the vectors $\varphi \left(z\right),{\;\varphi }^{o}$ of the problem (Equation (12)), taking into account the weight of the criteria u_i_:

(14) $$\rho \left(\phi \left(z\right),\phi^{o}\right)=\sqrt{\overset{k}{\underset{i=1}{\sum }}u_{i}\left(f_{i}\left(z\right)-f_{i}\left(z^{i0}\right)\right)}$$

**Figure 19:**
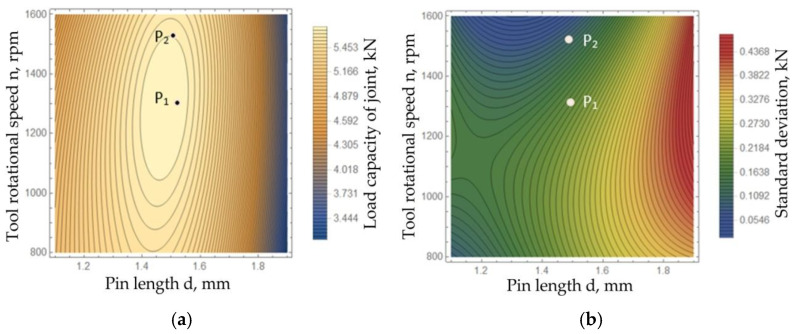
Solution of the problem of multi-criteria optimisation for a welding speed of x_2_ = 200 mm/min: (**a**) contour plot of the regression function of load capacity of joint, (**b**) contour plot of the regression function of the standard deviation of the results.

During the analysis, the same weight values (u_i_ = 0.5) were adopted. The function in Equation (12) reached a minimum with a tool length of 1.46 mm, welding speed of 200 mm/min, and tool rotational speed of 1517 rpm. These parameters were found to be optimal considering both optimisation criteria. The application of the parameters presented provided the possibility of obtaining a load capacity of the joint with an average value of 5.65 kN (point P_2_ in Figure 19a) and with a standard deviation of 0.09 kN (point P_2_ in Figure 19b). The indication of these parameters as optimal in terms of ensuring high load capacity while maintaining a low value of the standard deviation distinguishes this work from similar works in which the issue of optimising the parameters of the welding process was discussed in terms of the selection of parameters ensuring only the highest strength of joints.

The compromise solution thus obtained was more advantageous than the partial solution f_1_(z^1o^), which guaranteed the possibility of obtaining a higher load capacity of the joint of 5.71 kN (point P_1_ in Figure 19a), but with a standard deviation of 0.21 kN (point P_1_ in Figure 19b). If the welding process is not disturbed, it can be assumed that the scatter of the results will be subject to a normal distribution. The minimum value of the force that could be obtained for the parameters from the partial solution ($\bar{F}-3\sigma$) in this case amounted to 5.08 kN. The use of the parameters obtained as a result of the multi-criteria optimisation allowed a minimum load capacity of joints of 5.38 kN to be obtained with a much greater stability of the results.

## 4. Conclusions

In this article, the multi-criteria optimisation of FSW parameters with regard to the maximum load capacity of the joint and the minimum value of the dispersion of the load capacity has been carried out. The following conclusions can be drawn based on the results of the experimental research and optimisation analysis:

- An increase in welding speed at the value of the pin length considered caused a decrease in the load capacity of the joint as well as a significant increase in the dispersion of the results.
- Among the combinations of parameters that were considered, the lowest load capacity was found in joints made using the following parameters: pin length d = 1.5 mm, welding speed f = 400 mm/min, and tool rotational speed n = 800 rpm.
- The metallographic analysis also made it possible to identify a typical defect for FSW joints, namely a cold lap defect on the retracting side of the weld. As in the case of the hook defect, there was no metallic continuity in the cold lap area.
- The shear failure mode was the most frequently observed mechanism of the destruction of joints made at the smallest pin length that was considered (d = 1.1 mm).
- Regardless of the tool rotational speed adopted, the highest strength of the joint was observed for the pin length d = 1.5 mm. After this value was exceeded, all the characteristics showed a trend of decreasing joint strength with increasing pin length.
- For the joints processed with a welding speed of 400 mm/min, the load capacity of the joint increased with the increasing tool rotational speed.
- At lower values of tool rotational speed, the standard deviation of the process increased with an increase in pin length.
- The use of the parameters obtained as a result of the multi-criteria optimisation allowed a minimum load capacity of joints of 5.338 kN to be obtained with a much greater stability of the results; the lowest value of the standard deviation was obtained here (SD = 0.043 kN).
- As a result of the multi-criteria optimisation, taking into account both optimisation criteria, the optimal parameters were welding speed f = 200 mm/min, tool rotational speed n = 1517 rpm, and pin length d = 1.46 mm; for these parameters, the load capacity of the connection was demonstrated to be 5.65 kN and standard deviation SD = 0.09 kN.
- The highest load capacity (5.527 kN) was found in joints made using the following parameters: pin length d = 1.5 mm, welding speed f = 200 mm/min, and tool rotational speed n = 1200 rpm.

## Figures and Tables

**Table 1:** Chemical composition of the EN AW-2024-T3 aluminium alloy (wt.%).

Cu	Mg	Mn	Si	Fe (max.)	Zn (max.)	Ti	Cr	Others		Al
								Each	Total	
3.8–4.9	1.2–1.8	0.3–0.9	0.5	0.5	0.25	0.15	0.1	0.05	0.015	balance

**Table 2:** Basic mechanical properties of the EN AW-2024-T3 aluminium alloy [44].

Yield Stress R_p0.2_, MPa	Ultimate Tensile Stress R_m_, Mpa	Elongation A_5_, %	Hardness, HB
250–290	360–425	12–14	104–123

**Table 3:** The welding parameters used in the DoE matrix assigned to the DoE level (−1, 0, +1).

DoE Level	Pin Length d, mm	Welding Speed f, mm/min	Tool Rotational Speed n, rpm
−1	1.1	200	800
0	1.5	300	1200
+1	1.9	400	1600

**Table 4:** Standardised effects of the welding parameters on the tensile strength (S—shear failure mode, T—tensile failure mode).

Variant No.	Pin Length d, mm	Welding Speed f, mm/min	Tool Rotational Speed n, rpm	Failure Mode	Tensile Force (Sample A), kN	Tensile Force, (Sample B), kN	Tensile Force (Sample C), kN	Average Tensile Force, kN	Standard Deviation of Tensile Force, kN
1	1.1	200	800	S	4.888	4.829	4.684	4.801	0.085
2	1.1	200	1200	S	5.001	4.973	4.718	4.898	0.127
3	1.1	200	1600	S	4.651	4.783	4.719	4.717	0.054
4	1.1	300	800	S	5.002	4.898	5.048	4.983	0.062
5	1.1	300	1200	S	5.075	4.982	5.144	5.067	0.067
6	1.1	300	1600	S	3.906	4.879	5.015	4.600	0.493
7	1.1	400	800	S	5.145	4.826	4.654	4.875	0.204
8	1.1	400	1200	S	5.045	4.905	4.878	4.943	0.073
9	1.1	400	1600	S	5.282	5.319	5.213	5.271	0.044
10	1.5	200	800	T	5.395	5.328	5.291	5.338	0.043
11	1.5	200	1200	T	5.621	5.641	5.412	5.527	0.103
12	1.5	200	1600	T	5.408	5.209	5.440	5.352	0.102
13	1.5	300	800	T	4.704	4.120	4.678	4.501	0.269
14	1.5	300	1200	T	5.001	4.882	5.197	5.027	0.130
15	1.5	300	1600	T	4.285	3.929	4.251	4.155	0.161
16	1.5	400	800	T	0.803	0.814	1.078	0.898	0.127
17	1.5	400	1200	T	2.390	1.985	2.342	2.239	0.181
18	1.5	400	1600	T	3.136	3.075	3.189	3.134	0.046
19	1.9	200	800	T	3.980	3.578	3.147	3.569	0.339
20	1.9	200	1200	T	3.849	2.943	3.377	3.390	0.369
21	1.9	200	1600	T	3.169	3.867	3.304	3.447	0.302
22	1.9	300	800	T	2.187	2.413	2.717	2.439	0.217
23	1.9	300	1200	T	2.882	2.757	3.169	2.936	0.172
24	1.9	300	1600	T	3.358	2.355	3.259	2.991	0.451
25	1.9	400	800	T	0.880	0.986	0.894	0.921	0.047
26	1.9	400	1200	T	1.655	1.696	1.513	1.621	0.078
27	1.9	400	1600	T	2.628	3.247	2.669	2.848	0.283

**Table 5:** Values of the coefficients of the regression equations W_F_(x) and W_σ_(x).

Coefficient	W_F_(x)	W_σ_(x)
$b_{0}$	−17.4188	−2.41201
$b_{1}^{\left(1\right)}$	21.4528	1.62391
$b_{2}^{\left(1\right)}$	0.12861	−0.00248
$b_{3}^{\left(1\right)}$	−0.00091	0.004926
$b_{12}^{\left(1\right)}$	−0.24367	−0.001336
$b_{13}^{\left(1\right)}$	0.00720	−0.004807
$b_{23}^{\left(1\right)}$	−0.000049	3.5818 × 10^−6^
$b_{12}^{\left(2\right)}$	0.08203	−0.00211
$b_{21}^{\left(2\right)}$	−0.000023	9.964 × 10^−6^
$b_{13}^{\left(2\right)}$	0.00197	0.001643
$b_{12}^{\left(2\right)}$	-	−3.006 × 10^−8^
$b_{23}^{\left(2\right)}$	-	6.0817 × 10^−9^
$b_{11}^{\left(2\right)}$	8.3457	0.84206
$b_{22}^{\left(2\right)}$	0.000278	0.000013
$b_{33}^{\left(2\right)}$	1.51 × 10^−6^	−1.688 × 10^−6^
$b_{111}^{\left(3\right)}$	−7.1218	−0.40246
$b_{222}^{\left(3\right)}$	−4.34 × 10^−7^	-
$b_{333}^{\left(3\right)}$	-	-

## Data Availability

The data presented in this study are available on request from the corresponding author.
